# PICOT binding to chromatin-associated EED negatively regulates cyclin D2 expression by increasing H3K27me3 at the *CCND2* gene promoter

**DOI:** 10.1038/s41419-019-1935-0

**Published:** 2019-09-17

**Authors:** Pinakin Pandya, Minesh Jethva, Eitan Rubin, Ramon Y. Birnbaum, Alex Braiman, Noah Isakov

**Affiliations:** 1 The Shraga Segal Department of Microbiology, Immunology and Genetics, Faculty of Health Sciences and the Cancer Research Center, Ben Gurion University of the Negev, P.O.B. 653, 84105 Beer Sheva, Israel; 2 Department of Life Sciences, Ben-Gurion University of the Negev, 84105 Beer-Sheva, Israel

**Keywords:** Immunology, Molecular biology

## Abstract

Protein kinase C (PKC)-interacting cousin of thioredoxin (PICOT; also termed glutaredoxin 3 (Grx3; Glrx3)) is a ubiquitous protein that can interact with the embryonic ectoderm development (EED) protein via each of its two C-terminal PICOT/Grx homology domains. Since EED is a Polycomb-Group protein and a core component of the polycomb repressive complex 2 (PRC2), we tested the involvement of PICOT in the regulation of PRC2-mediated H3 lysine 27 trimethylation (H3K27me3), transcription and translation of selected PRC2 target genes. A fraction of the cellular PICOT protein was found in the nuclei of leukemia cell lines, where it was associated with the chromatin. In addition, PICOT coimmunoprecipitated with chromatin-residing EED derived from Jurkat and COS-7 cell nuclei. PICOT knockdown led to a reduced H3K27me3 mark and a decrease in EED and EZH2 at the *CCND2* gene promoter. In agreement, PICOT-deficient T cells exhibited a significant increase in *CCND2* mRNA and protein expression. Since elevated expression levels of PICOT were reported in several different tumors and correlated in the current studies with decreased transcription and translation of the *CCND2* gene, we tested whether this opposite correlation exists in human cancers. Data from the Cancer Genome Atlas (TCGA) database indicated statistically significant negative correlation between *PICOT* and *CCND2* in eight different human tumors where the highest correlation was in lung (*p* = 8.67E−10) and pancreatic (*p* = 1.06E−5) adenocarcinoma. Furthermore, high expression of *PICOT* and low expression of *CCND2* correlated with poor patient survival in five different types of human tumors. The results suggest that PICOT binding to chromatin-associated EED modulates the H3K27me3 level at the *CCND2* gene promoter which may be one of the potential mechanisms for regulation of cyclin D2 expression in tumors. These findings also indicate that a low *PICOT/CCND2* expression ratio might serve as a good predictor of patient survival in selected human cancers.

## Introduction

Protein kinase C (PKC)*-*interacting cousin of thioredoxin (PICOT, also termed glutaredoxin 3 (Grx3; Glrx3)), was discovered in the year 2000 in a search for PKCθ binding proteins^1^. PICOT possesses three evolutionary conserved domains including an N-terminal thioredoxin (Trx) homology domain and a tandem repeat of a PICOT/glutaredoxin (Grx) homology domain^2,3^. However, in contrast to Trx and Grx, which possess two redox-active cysteines at their catalytic site, PICOT possesses only a single cysteine residue in the putative catalytic region of each of the three domains and is therefore devoid of the classical Trx or Grx catalytic activities.

While the exact biological role of PICOT has not yet been resolved, its critical role in embryonic development has been validated in PICOT deficient mice that showed growth retardation and morphological defects during development, leading to death at ~12.5 days post coitum^4,5^.

PICOT is a ubiquitously expressed protein that resides predominantly in the cytoplasm^6^. However, it is also found in cell nuclei^6^, and its subcellular distribution at a given time depends on the cell type and the stage of activation/differentiation^7^.

PICOT regulates the c-Jun N-terminal kinase (JNK)/AP-1 and NF-κB pathways in T cells, where it colocalizes with PKCθ^1^. Its expression is upregulated in cardiac muscles in response to hypertrophic stimuli where PICOT attenuates the pressure overload-induced cardiac hypertrophy and increases ventricular function and cardiomyocyte contractility^5,8^. Further studies demonstrated that PICOT plays a role in cellular iron metabolism and biogenesis of Fe/S proteins and hemoglobin maturation^9,10^. PICOT expression levels are augmented in mitogen-stimulated or antigen-stimulated T cells^11^, as well as in various tumor cell lines^11^. In addition, high expression levels of PICOT were observed in vivo in a variety of cancers, including Hodgkin’s lymphoma^11^ and breast^7^, colon, and lung carcinoma^12^.

Utilization of a full-length human *PICOT* cDNA in a yeast two-hybrid screen of a Jurkat T cell cDNA library revealed that PICOT interacts with the embryonic ectoderm development (EED) protein^13^. PICOT interaction with EED was verified in various human cell lines and reconfirmed using GST pull-down assays, reciprocal coimmunoprecipitation and immunofluorescence imaging^13^. Binding of PICOT to EED is mediated by each of its two C-terminal PICOT/Grx homology domains^13^.

EED is a member of the Polycomb-Group (PcG) proteins^14,15^ that are critical for chromatin remodeling and epigenetic gene silencing^16^. EED serves as a core component of the polycomb repressive complex 2 (PRC2) which catalyzes histone H3 trimethylation on lysine 27 (H3K27me3), a mark of transcriptional repression of multiple genes^17^.

Based on the above information, we hypothesized that PICOT interaction with EED might have an impact on transcriptional processes of PRC2 target genes. Initial studies supported this hypothesis by demonstrating that PICOT knock-down in Jurkat T cells led to a reduced H3K27me3 at the PRC2 target gene, *myelin transcription factor 1* (*MYT1*)^13^.

In the present study, we further analyzed the potential involvement of PICOT in the regulation of PRC2-mediated H3K27 trimethylation. We also analyzed the effect of PICOT on the transcription and translation of selected PRC2 target genes and tested the potential relevance of this regulation to cancer progression.

## Results

### Nuclear PICOT partially colocalizes with EED in leukemia cells

**Fig. 1:**
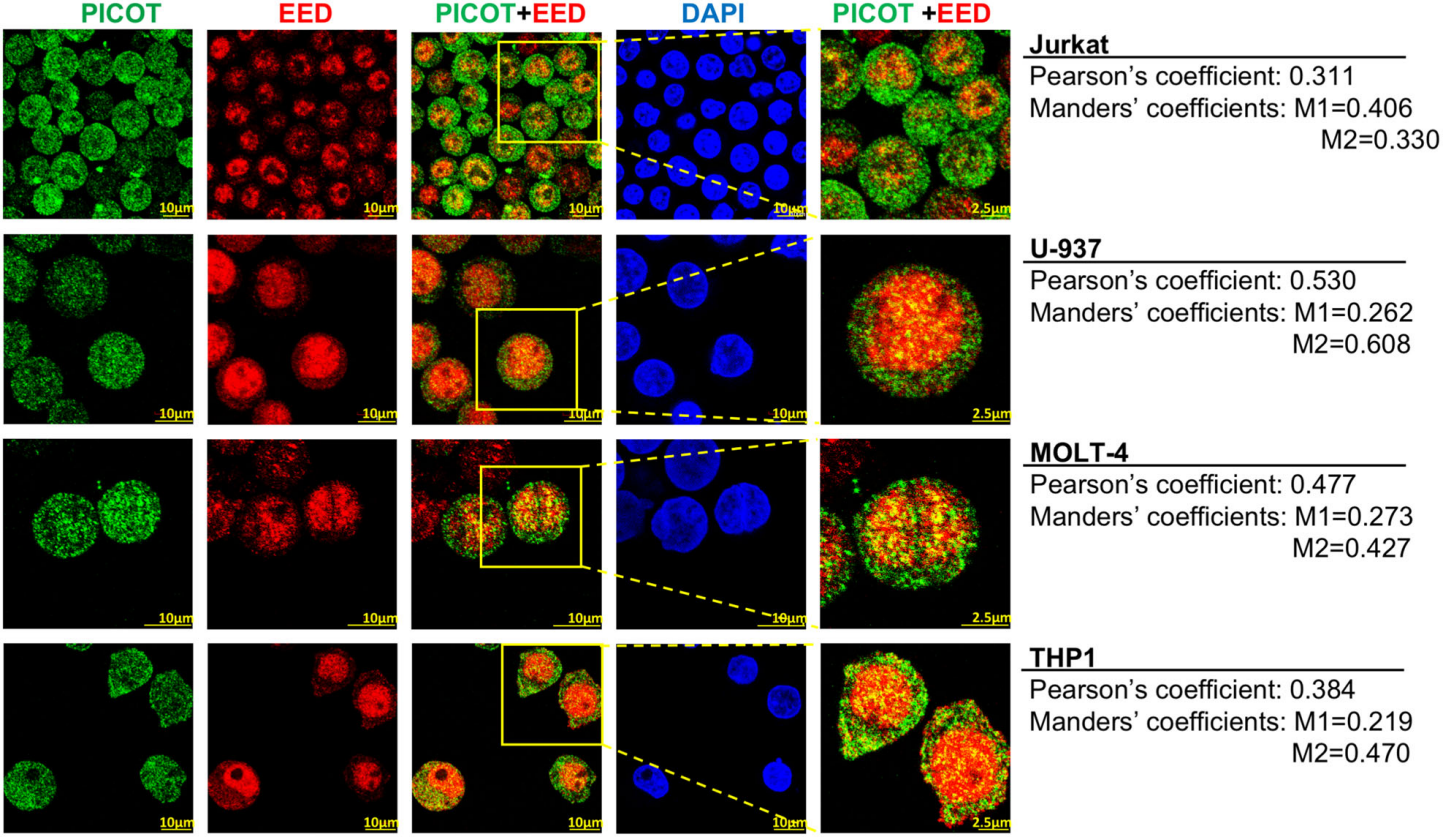
Nuclear PICOT partially colocalizes with EED in leukemia cells. Jurkat, U-937, MOLT4 and THP1 cells (1.5 × 10^6^/group) were seeded on poly-L-lysine-coated 8 well µ-slide (ibidi Ltd.) and deposited by centrifugation at 1200 rpm for 5 min. The cells were fixed, permeabilized and incubated with a mixture of mouse anti-PICOT mAbs and rabbit anti-EED polyclonal Abs for 1 h at room temperature. The cells were then stained using Cy3-conjugated anti-mouse Abs and Cy5-conjugated anti-rabbit Abs, plus nuclei counterstain with DAPI (1 µg/ml in PBS) for 1 h in the dark, at room temperature. The cells were then analyzed using a confocal microscope (Olympus Flouview 1000 laser scanning confocal microscope). PICOT-EED colocalization is demonstrated in a color overlay panel, and a selected area marked by a red box that was magnified is shown in the right panel. Comparative quantification of PICOT-EED colocalization was performed using the ImageJ colocalization *JACoP imageJ* plugin^18^. Pearson’s coefficient value and Manders’ coefficient values (M1 = red overlap with green; M2 = green overlap with red) are indicated on right for each panel

To analyze whether PICOT colocalizes with nuclear EED in leukemia cells, we performed immunofluorescence staining of PICOT and EED in four different human leukemia cell lines. Laser scanning confocal microscopy of the stained cells reconfirmed the predominant nuclear localization of EED and demonstrated that PICOT resides in both the cytoplasm and nucleus (Fig. 1). Interestingly, overlay images revealed partial colocalization of the two proteins predominantly in the nuclei of all cell lines (Fig. 1), suggesting that PICOT and EED might function together in the nucleus. The extent of PICOT-EED colocalization in each of the cell lines was calculated using the ImageJ colocalization JACoP imageJ plugin^18^ and values are shown in Fig. 1.

Specificity of the anti-PICOT Abs was verified by the lack of immunofluorescence staining of PICOT-deficient Jurkat T cells (Jurkat.1A) (not shown), whereas the specificity of the anti-EED Abs was demonstrated on EED-deficient Jurkat T cells obtained by transfection of EED-specific small interfering (si)RNA^19^.

### PICOT reside in the chromatin fraction of tumor cell lines

As the nuclear PICOT colocalizes with EED, a core component of PRC2, which associates with chromatin and maintains its repressive state^13^, we tested whether PICOT can also associate with chromatin.

**Fig. 2:**
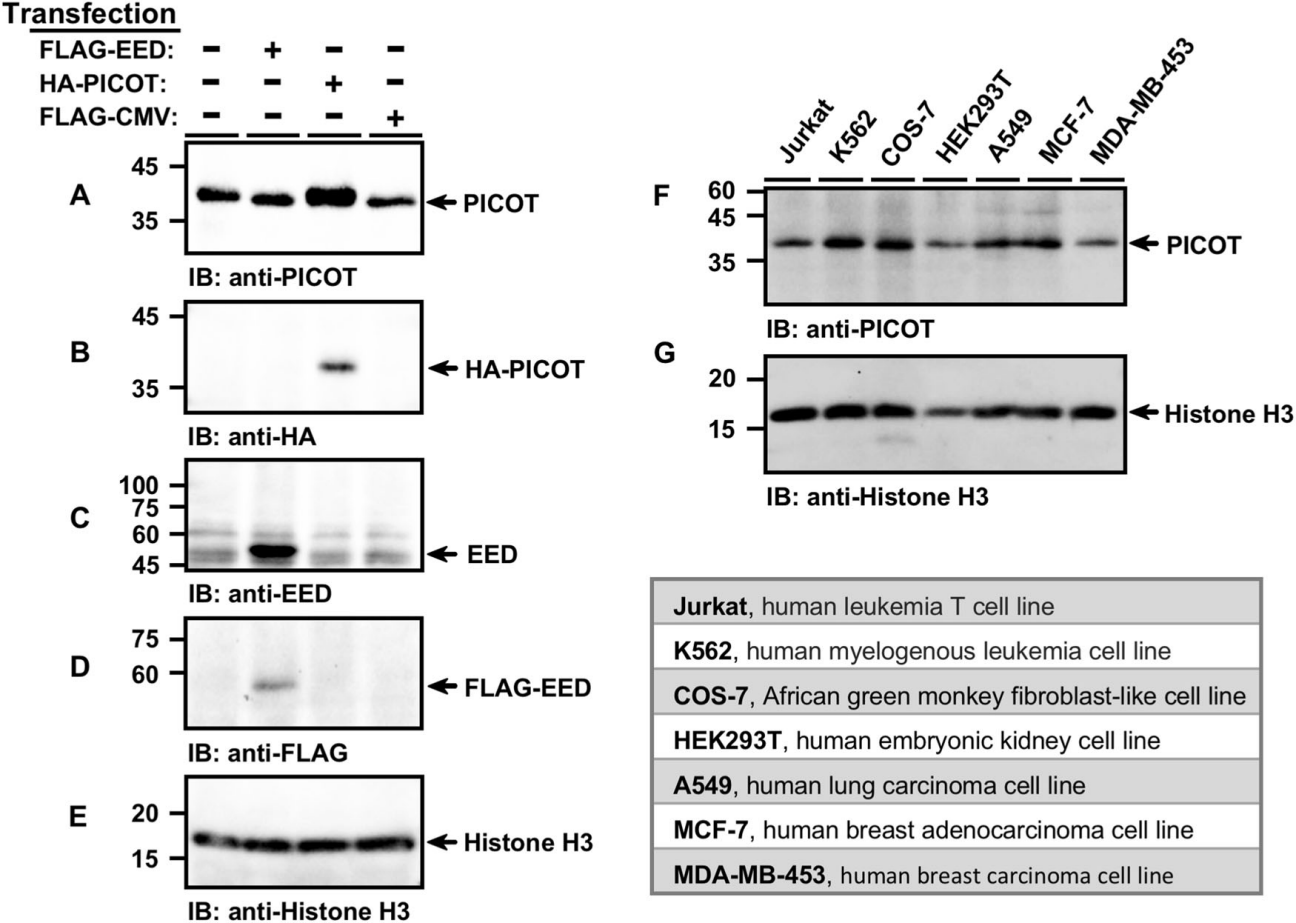
PICOT reside in the chromatin fraction of tumor cell lines. COS-7 cells were transiently transfected with the indicated expression vectors using the PEI reagent. Chromatin lysates from transfected and untransfected COS-7 cells were prepared using the protein-protein ChIP protocol, boiled, and subjected to SDS-PAGE (5 µg/lane) on two parallel 12.5% gels under reducing conditions. Proteins were then electroblotted onto two parallel nitrocellulose membranes that were immunoblotted with either mouse anti-PICOT mAbs (**a**), or rabbit anti-HA polyclonal Abs (**b**), followed by development using the immunoperoxidase ECL detection system and autoradiography. The membranes were then immunoblotted with rabbit anti-EED Abs (**c**), mouse anti-FLAG mAbs (**d**) and mouse anti-Histone H3 Abs (**e**), which served as a protein loading control. In a similar experiment, chromatin lysates were prepared from seven different cell lines and samples were immunoblotted with mouse anti-PICOT mAbs (**f**) and mouse anti-Histone H3 mAbs (**g**). The origin of the cell lines is indicated in the table below

COS-7 cells were transiently transfected with *HA-PICOT*, *FLAG-EED*, or a control *FLAG-CMV* expression vector followed by chromatin isolation, as indicated in the standard chromatin immunoprecipitation (ChIP) assay. Western blot analysis revealed that the chromatin fraction included both endogenous and heterologous PICOT proteins (Fig. 2a, b), as well as endogenous and heterologous EED proteins (Fig. 2c, d).

To further analyze whether the presence of PICOT in chromatin lysates is a general phenomenon, we isolated chromatin from seven different cell lines followed by SDS-PAGE fractionation. Western blot analysis revealed that PICOT resides in the chromatin fraction of all tested cell lines (Fig. 2f), suggesting a functional role for nuclear PICOT in chromatin regulation.

### PICOT associate with chromatin residing EED

**Fig. 3:**
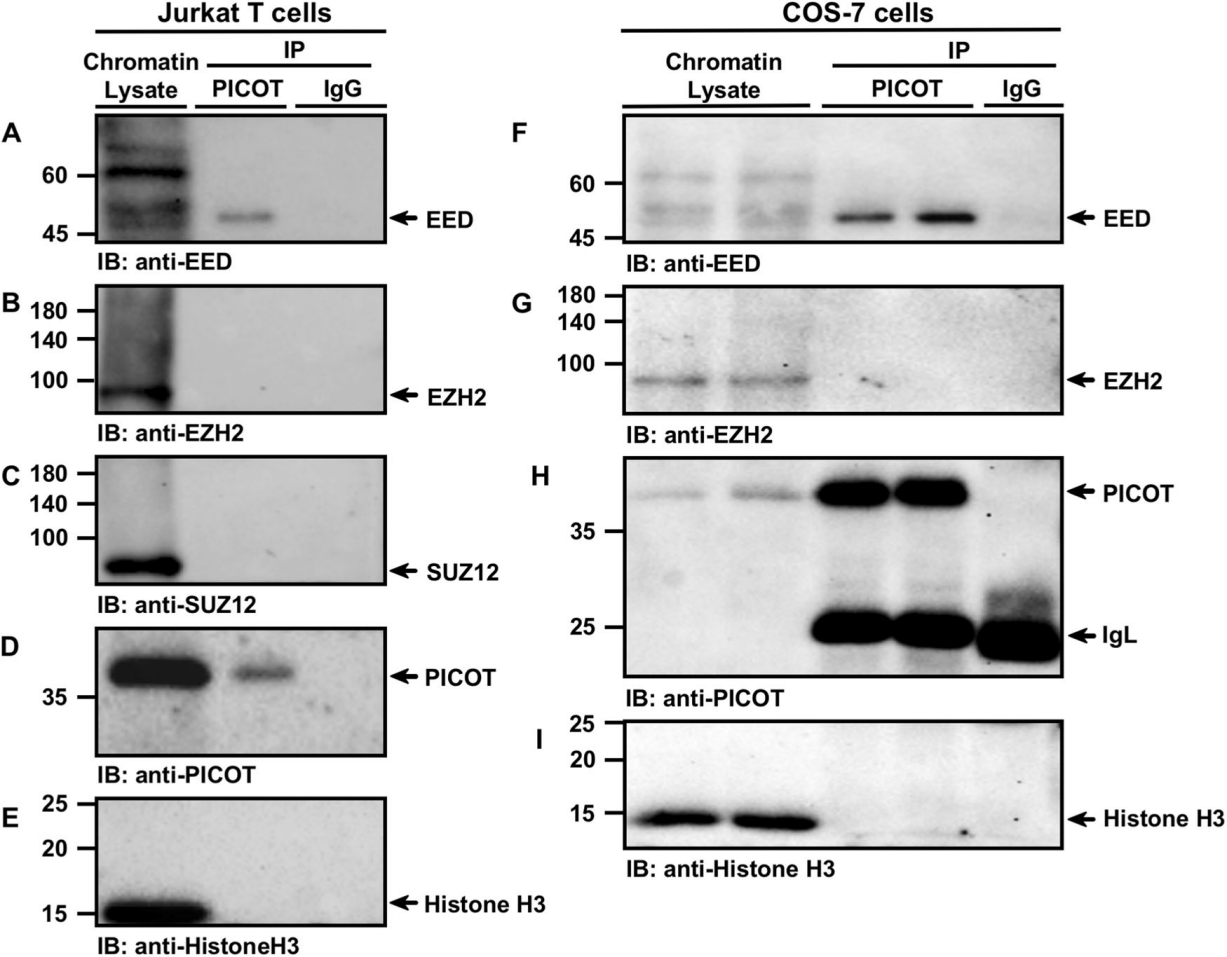
PICOT-EED interaction co-exists in the chromatin compartment. Jurkat and COS-7 cells (20 × 10^6^/group) were fixed in 1% formaldehyde for 10 min at room temperature and chromatin lysates were prepared using the protein-protein ChIP protocol. The chromatin samples were precleared on protein G-sepharose beads, incubated overnight at 4 °C with protein G sepharose bead-immobilized mouse anti-PICOT mAbs or mouse normal IgG (10 × 10^6^ cell equivalent per group). Proteins were then eluted from the beads by resuspension of the samples in β-mercaptoethanol-containing sample buffer (180 µl) and boiling for 30 min. Following centrifugation, supernatants (1.5 × 10^6^ cell equivalent in 20 µl) were subjected to SDS-PAGE on 10% gel under reducing conditions. Chromatin lysates (5 µg/lane) were boiled and electrophoresed in parallel. Proteins were then electroblotted onto nitrocellulose membranes that were sequentially immunoblotted with rabbit anti-EED polyclonal Abs (**a**, **f**) and rabbit anti-EZH2 mAbs (**b**, **g**). A parallel membrane was sequentially immunoblotted with rabbit anti-SUZ12 mAbs (**c**), mouse anti-PICOT mAbs (**d**, **h**) and mouse anti-histone H3 mAbs (**e**, **i**). Protein bands were then visualized using the immunoperoxidase ECL detection system and autoradiography. Immunoprecipitation using IgG mAbs served as a negative control. The position of specific protein bands is indicated by arrows. Results are representative of three independent experiments. IP, immunoprecipitation; IB, immunoblot

To test whether PICOT interacts with chromatin-associated EED, we performed specific co-immunoprecipitation on chromatin fraction of Jurkat T cell lysates using PICOT-specific Abs. We found that EED coimmunoprecipitated with PICOT, while normal IgG that served as a control did not immunoprecipitate EED (Fig. 3a). In contrast, anti-PICOT Abs did not coimmunoprecipitate other PRC2 core components, such as EZH2 (Fig. 3b) or SUZ12 (Fig. 3c).

As a technical control, PICOT immunoprecipitates that were immunoblotted with anti-PICOT Abs showed efficient recovery of PICOT proteins from chromatin lysates (Fig. 3d). Furthermore, these results were repeated using COS-7 cells, where EED (Fig. 3f), but not EZH2 (Fig. 3g) coimmunoprecipitated with PICOT.

To further substantiate these findings, we performed a reciprocal coimmunoprecipitation on Jurkat T cell chromatin lysates and found that anti-EED-Abs coimmunoprecipitated both PICOT (Fig. 1sA) and EZH2 (Fig. 1sB). The reciprocal coimmunoprecipitation studies (Fig. 1s) suggest that the chromatin-associated EED can interact with both PICOT and EZH2. However, the PICOT-associated EED does not bind EZH2 (Fig. 3), suggesting that PICOT binds chromatin-associated EED which is free of the other PRC2 core proteins.

### Knockdown of PICOT results in reduced association of H3K27me3, EED and EZH2 at the *CCND2* gene promoter

Since PICOT association with EED occurs at the chromatin level, we tested whether this interaction can alter the PRC2-mediated H3K27me3 levels at PRC2 target genes. Using ChIP-qPCR, we compared the levels of trimethyl H3K27 at selected target genes in chromatin lysates of control vs. PICOT-deficient Jurkat T cells.

**Fig. 4:**
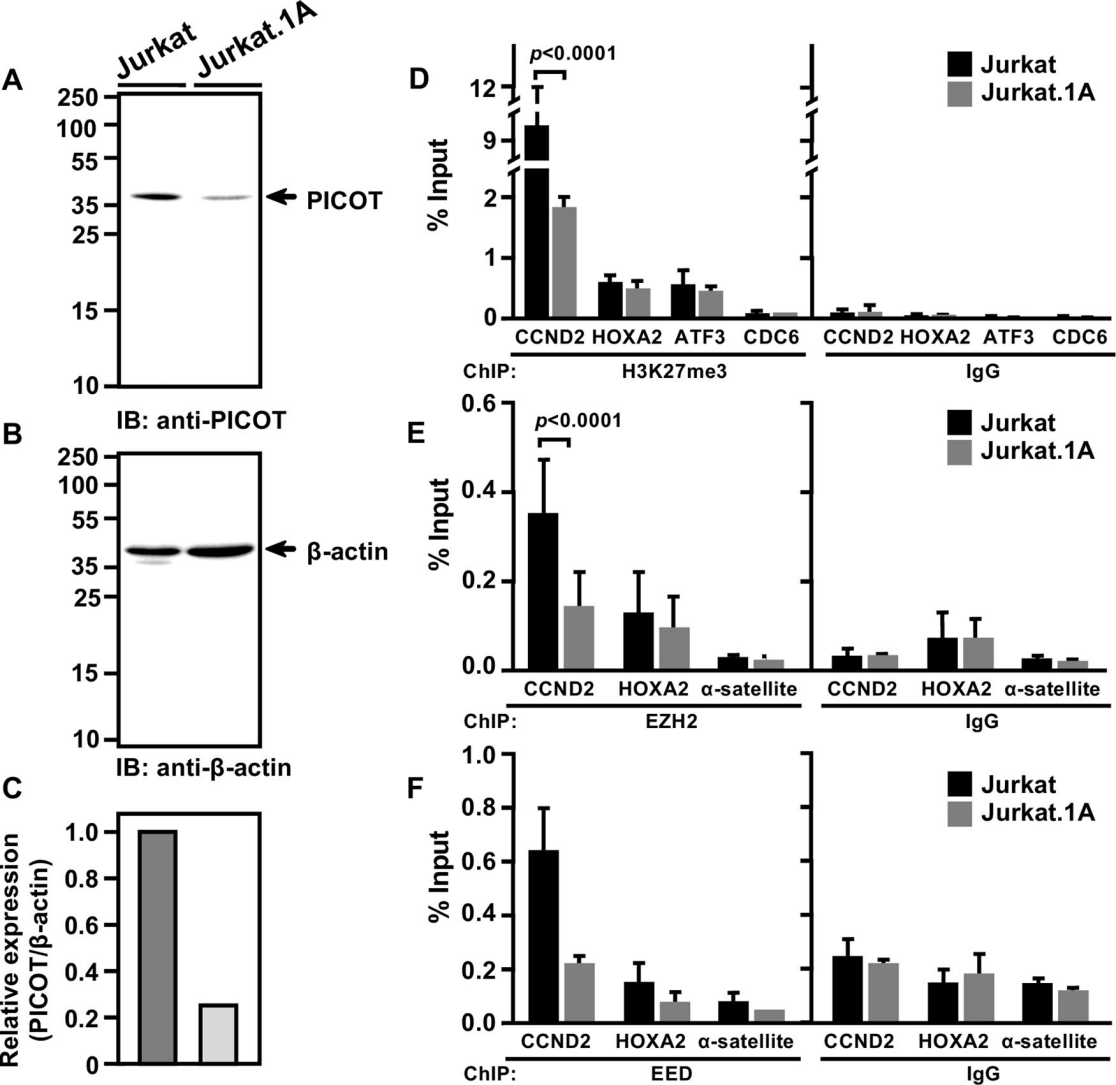
Knockdown of PICOT results in reduced association of H3K27me3, EED, and EZH2 with the *CCND2* gene promoter. Jurkat and PICOT-deficient Jurkat.1A cells were resuspended in lysis buffer on ice (30 min) and centrifuged. Nuclear free supernatants were collected, boiled, and subjected to SDS-PAGE on 10% gel under reducing conditions (12.5 µg/lane). Proteins were electroblotted onto a nitrocellulose membrane and sequentially immunoblotted with mouse anti-PICOT (**a**) and mouse anti-β-actin mAbs (**b**) followed by the immunoperoxidase ECL detection system and autoradiography. A bar graph shows the relative expression of PICOT in the two cell lines (**c**). For ChIP-qPCR assay, Jurkat and Jurkat.1A cells (20 × 10^6/^group) were fixed using 1% formaldehyde (10 min at room temperature), washed twice in cold PBS, and then lysed by resuspension in lysis buffer for 10 min on ice. Nuclear pellets, obtained by centrifugation (5 min, 5000 rpm) were resuspended in nuclear lysis buffer, sonicated, centrifuged, and precleared on protein G-sepharose beads. The chromatin lysates were incubated with protein-G sepharose bead-immobilized rabbit anti-H3K27me3 mAbs (**d**), rabbit anti-EZH2 mAbs (**e**), rabbit anti-EED polyclonal Abs (09–774) (**f**), or rabbit anti-IgG Abs, as a control. After overnight incubation on a rotator, the beads were washed sequentially with low-salt buffer, high-salt buffer, LiCl buffer and tris-EDTA buffer. Precipitated DNA was eluted from immune complexes and in parallel, DNA was eluted from the crude sonicated chromatin lysates. Isolated DNA was tested for the presence of several target genes using appropriate sets of primers in RT-PCR. The relative level of protein enrichment following immunoprecipitation with specific Abs vs. isotype control (IgG) at different gene loci in Jurkat vs. Jurkat.1A was determined as a percentage of the total input signal. Bar graph represents the mean ± standard deviation of the calculated values (**d**–**f**). The ChIP assay was biologically repeated twice and the qPCR analyses were performed in triplicates. *CDC6* and *α-satellite* were used as non-target genes and ChIP using IgG served as a negative control for immunoprecipitation. Two-way ANOVA was calculated using GraphPad Prism 7 software to determine the level of significance between two cell lines

We initially tested the expression levels of PICOT in the two cell lines and found that PICOT-deficient Jurkat T cells (Jurkat.1A) express ~5-fold lower levels of PICOT compared to the control Jurkat cells (Fig. 4a).

Next, we performed ChIP-qPCR using H3K27me3 and IgG isotype (as a control) and analyzed the H3K27me3 enrichment at selected genomic regions that include three PRC2 target genes: *Cyclin*-D2 (*CCND2*), *Homeobox A2* (*HOXA2*) and *Activating transcription factor 3* (*ATF3*), as well as *Cell division cycle 6* (*CDC6*), a non-PRC2 target gene that serves as a negative control. We found a ~3.5-fold significant H3K27me3 enrichment (*p* < 0.0001) on the promoter of *CCND2* in Jurkat T cells when compared to the PICOT-deficient cells (Fig. 4d). In contrast, no H3K27me3 enrichment was observed at *HOXA2* and *ATF3*, or at the PRC2 non-target gene, *CDC6* (Fig. 4d).

The fact that DNA enrichment was not general, and observed at selected genes (*CCND2*) was not surprising, since desilence of selected PRC2 target genes has already been reported following knockdown of genes of various PRC2-associated proteins^20^.

Furthermore, when the ChIP-qPCR was optimized for the PRC2 components, EZH2 and EED, the percentage input value for the PRC2 target genes was significantly higher when using either anti-EED (Fig. 2sB) or anti-EZH2 Abs (Fig. 2sC), compared to isotype control.

The percentage input values for the PRC2 non-target genes, *α-satellite*, were similar when either anti-EED (Fig. 2sB) or anti-EZH2 Abs (Fig. 2sC) were compared to isotype control.

Since the trimethylation of H3K27 is catalyzed by EZH2^20,21^ and further propagated by the help of EED^22,23^, we further tested whether PICOT knockdown might effect EZH2 and EED presence at PRC2 target genes.

Using ChIP-qPCR with anti-EZH2 Abs, we revealed a ~2.5-fold lower enrichment of EZH2 at the *CCND2* promoter in PICOT deficient cells (Fig. 4e).

Moreover, ChIP-qPCR with anti-EED Abs showed a ~3-fold lower enrichment of EED at the *CCND2* promoter in PICOT deficient cells, compared to control Jurkat cells (Fig. 4f). In both EZH2 and EED ChIP analyses, no enrichment was observed at *HOXA2* and *α-satellite* in PICOT deficient cells (Fig. 4e, f).

Thus, PICOT associates with EED and promotes the trimethylation of H3K27 on the *CCND2* promoter, and might therefore have an effect on the expression of *CCND2*.

To test whether PICOT might be directly associated with the CCND2 promoter region we performed ChIP-qPCR using either anti-PICOT mAbs, to immunoprecipitate the endogenous PICOT protein from Jurkat cell chromatin lysates, or ChIP-grade anti-HA Abs to immunoprecipitatie a transiently expressed HA-PICOT from COS-7 cell chromatin lysates. Results demonstrated that the percentage input value for CCND2 using anti-PICOT mAbs (Fig. 3sA) or anti-HA Abs (Fig. 3sB) was not significantly different from that of the ‘beads only’ control group, suggesting that PICOT is not direclty associated with the CCND2 promoter.

Knock down of PICOT expression in Jurkat T cells increases cyclin D2 mRNA and protein expression levels

Since PRC2-mediated trimethyation of H3K27 at specific gene loci can negatively regulate gene transcripiton, we tested whether the lower levels of EED, EZH2, and H3K27me3 at the *CCND2* promoter in PICOT-deficient cells would also be reflected at the gene transcription and translation levels.

**Fig. 5:**
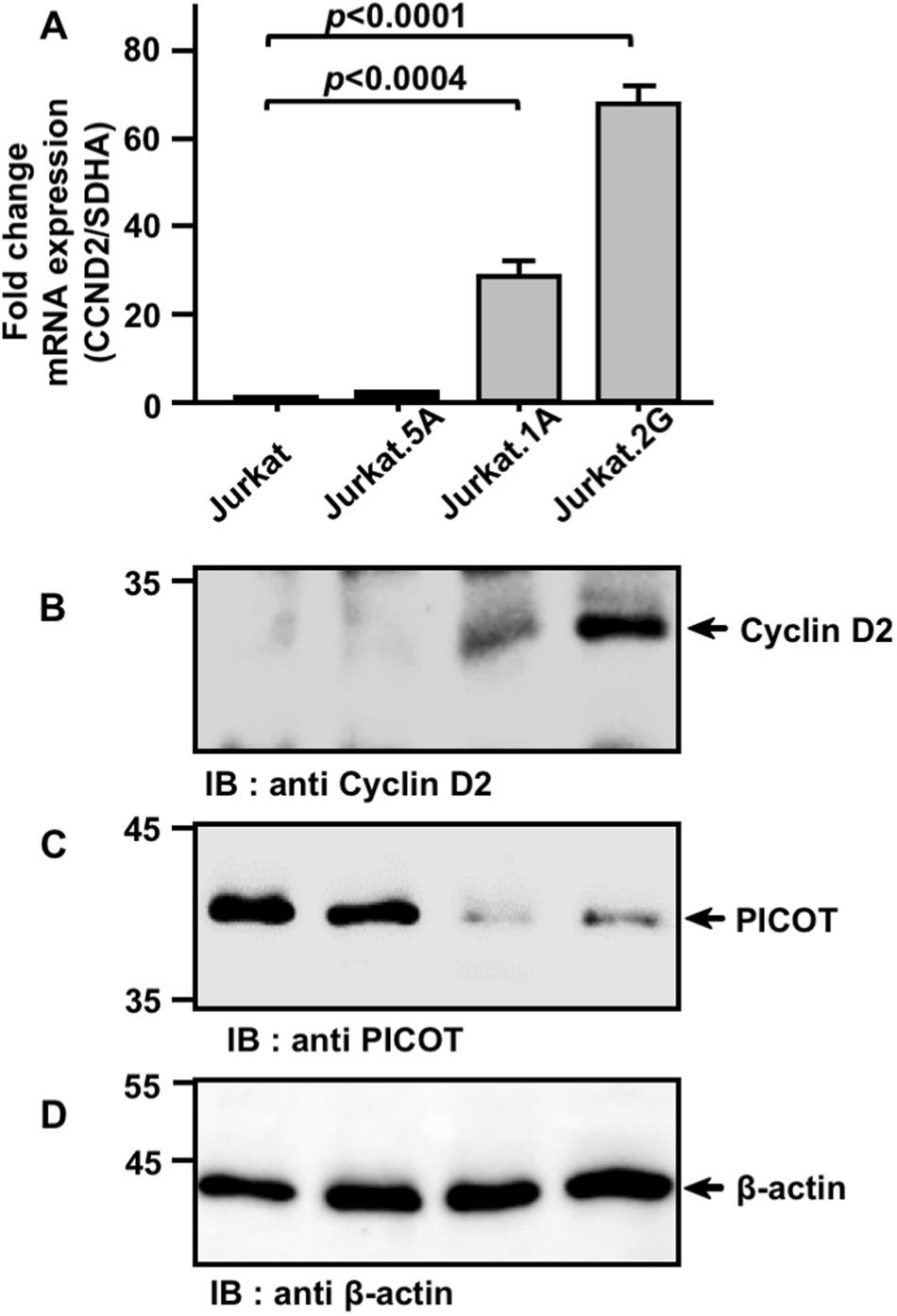
PICOT expression in Jurkat T cells negatively correlates with *CCND2* mRNA and protein expression. **a**. RNA was extracted from Jurkat cells, PICOT-deficient *sh-PICOT*-expressing Jurkat sublines (1A and 2G), and a control, *sc-PICOT* expressing Jurkat subline (5A). Total RNA from each sample (1 µg/sample) was isolated and served as a template for complementary DNA (cDNA) preparation using the Bio-RT^TM^ first strand cDNA synthesis kit (Bio-Lab) plus random hexamer primers. The cDNA generated during reverse transcription then served as template in the subsequent qRT-PCR that was carried out in the presence of *CCND2* or *SDHA* gene-specific primers and the double-strand DNA intercalating fluorescent dye, SYBR® green. The relative fold change in *CCND2* mRNA was determined by the ^ΔΔ^Ct method and calculated relative to the *SDHA* mRNA. The results are presented as a bar graph where mean ± standard error are indicated. Real-time PCR analyses were biologically repeated thrice with triplicate wells in each experiment. One-way ANOVA test was calculated using GraphPad Prism 7 software to determine the level of significance. **b**–**d**. For Western blot analysis, Jurkat and Jurkat-derived subclones (5 × 10^6^ cells/group) were resuspended in lysis buffer on ice (30 min), centrifuged and nuclear free supernatants were collected. Cell lysates (2.5 µg/lane) were boiled and subjected to SDS-PAGE on 10% gels under reducing conditions. Proteins were then electroblotted onto a nitrocellulose membrane, immunoblotted with rabbit anti-Cyclin D2 mAbs (**b**) followed by immunoperoxidase ECL detection system and autoradiography. Parallel membranes were immunoblotted with mouse anti-PICOT mAbs (**c**) and mouse anti-β-actin mAbs (**d**). The position of specific protein bands is indicated by arrows. Results are representative of three independent experiments

Using qRT-PCR, we tested the expression levels of *CCND2* in control and PICOT-deficient cells. The two PICOT-deficient sublines, Jurkat.1A and Jurkat.2G exhibited significantly higher expression levels of *CCND2* mRNA compared to the levels observed in control Jurkat T cells, or in Jurkat.5A subline, which expresses the control scrambled sc-PICOT DNA (Fig. 5a).

Increased *CCND2* mRNA levels in PICOT-deficient cells were not due to altered RNA-polymerase II (Pol II) levels at the CCND2 locus, since RNA-pol II ChIP data revealled similar levels of RNA-pol II at the CCND2 upstream promoter region in both wild type and PICOT-deficient Jurkat T cells (not shown).

Furthermore, determination of the levels of the *CCND2*-encoding protein, cyclin D2, in PICOT-deficient cells revealed high levels of protein expression in both PICOT-deficient cells, Jurkat.1A and Jurkat.2G, whereas cyclin D2 protein expression levels in Jurkat and Jurkat.5A were almost undetectable (Fig. 5b).

Western blot studies reconfirmed the lower levels of the PICOT protein in Jurkat.1A and Jurkat.2G cells (Fig. 5c), while the equal loading of lysate/lane was verified using anti-β-actin mAbs (Fig. 5d).

### *PICOT* expression levels negatively correlates with *CCND2* expression levels in human cancers

**Fig. 6:**
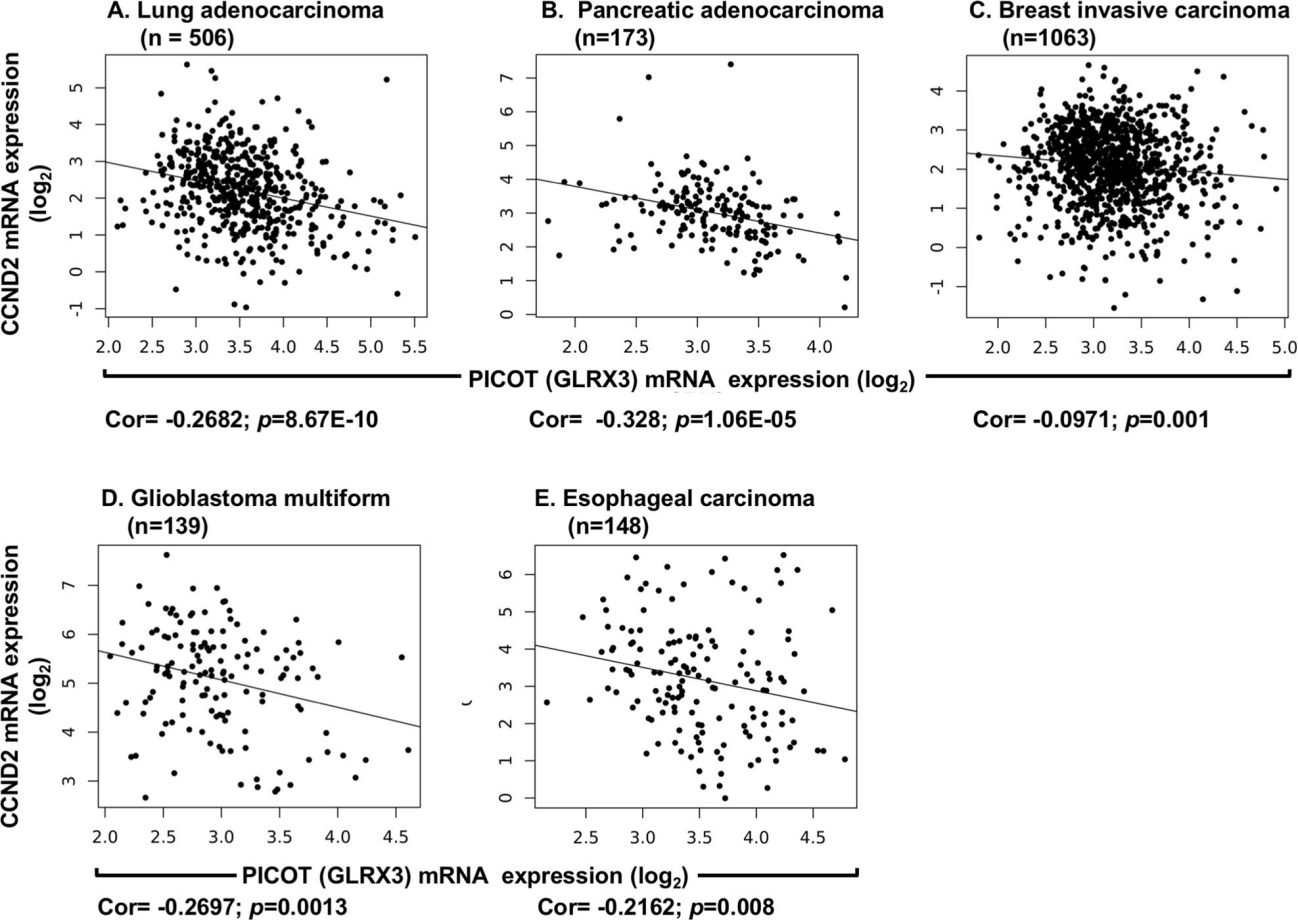
The expression levels of *PICOT* mRNA in five different types of human cancer negatively correlate with the *CCND2* gene mRNA expression levels. *PICOT* (*Glrx3*) and *CCND2* mRNA expression levels in five different types of human cancer tissues were downloaded from the TCGA using ISB Cancer Genomics Cloud. Outlier samples were removed by clipping the top and bottom deciles of each expression distribution. The correlation between *PICOT* and *CCND2* mRNA expression is illustrated as scatter plots where each dot represents a single cancer tissue sample. For each type of cancer, the total number of patients (N), the Pearson’s correlation value (Cor), and the *p* value are indicated

The current findings indicate that PICOT deficiency in Jurkat T cells correlates with hypomethylation of H3K27 at the *CCND2* promoter, which results in upregulation of *CCND2* gene transcription and translation, suggesting that PICOT might have a negative regulatory effect on the synthesis of cyclin D2. To analyze whether such a correlation might exist in human cancers, we downloaded data on *PICOT* and *CCND2* mRNA expression levels in 32 types of human cancers available at the Cancer Genome Atlas (TCGA) dataset. Using the Pearson’s correlation coefficient, we examined the extent of correlation between *PICOT* and *CCND2* in each cancer, after clipping the top and bottom decile of each measurement to avoid outlier effects (Fig. 6). The general tendency of negative correlation between the expression levels of *PICOT* and *CCND2* mRNA levels can be observed (Table Is).

However, a negative and significant correlation between *PICOT* and *CCND2* mRNA levels was only observed for 8 out of the 32 tumor types considered. Of these, the strongest correlation was observed in prostate adenocarcinoma (PRAD), lung adenocarcinoma (LUAD) and pancreatic adenocarcinoma (PAAD), with lower, but significant values in glioblastoma multiforme (GBM), breast invasive carcinoma (BRCA), gastric adenocarcinoma (STAD), esophageal carcinoma (ESCA) and ovarian cancer (OV). Subsequent analysis was conducted on 5 cancer types.

The correlation between *PICOT* and *CCND2* could be specific to cancer, reflecting abnormal regulation of the transformed cells, or could be universal and reflect basic cellular control mechanisms. To differentiate between these two hypotheses, we attempted to test the *PICOT-CCND2* correlation in normal tissues which are adjacent to the tumor (normal adjacent tissue; NAT), and compare them with the results obtained in the tumors. However, information on *PICOT* and *CCND2* mRNA expression levels in NAT samples was much less abundant and enabled statistical analysis only in BRCA (*n* = 96) and LUAD (*n* = 54). We found that, in contrast to the results observed in BRCA, where *PICOT* and *CCND2* mRNA levels exhibited a weak but significant negative correlation (Cor = −0.0971, *p* = 0.001), an opposite and non-significant correlation was observed in BRCA NAT (Cor = +0.03684, *p* = 0.721). The negative correlation between *PICOT* and *CCND2* mRNA expression levels that was observed in LUAD repeated also in LUAD NAT (Fig. 4s).

To analyze whether an opposite correlation exists between *PICOT* and *CCND2* mRNA levels in LUAD vs. LUAD NAT, and test the generality of this phenomenon, we used again data from the TCGA, and compared *PICOT* and *CCND2* mRNA expression levels in LUAD vs. LUAD NAT. The results (Fig. 5s) demonstrated a highly significant increase in *PICOT* mRNA expression in LUAD compared to LUAD NAT, and a highly significant decrease in *CCND2* mRNA expression in LUAD compared to LUAD NAT suggesting that increased *PICOT* and decreased *CCND2* mRNA levels can serve as a tumor marker in LUAD.

An independent analysis performed on T-ALL data from GSE62156, indicated a correlation of −0.3 (*p* = 0.016, *N* = 64; *t*-test on Pearson’s product moment correlation coefficient) between PICOT and CCND2 expression.

### A relatively high expression of PICOT and low expression of CCND2 correlates with poor patient survival in five different types of human cancers

Previous studies suggested that high expression levels of PICOT correlate with growth promotion of normal^11^ and cancer^11,12,24^ cells, whereas increased expression of Cyclin D2 negatively affects the growth of prostate cancer cells^25^. Since our data demonstrated a negative correlation between *PICOT* and *cyclin D2* mRNA expression levels in selected types of human cancers, we further analyzed the impact of such a correlation on the overall patients’ survival. Using publicly available TCGA datasets, the gene expression data and the patients’ clinical outcome were obtained for five types of cancer diseases, namely, lung adenocarcinoma (LUAD), breast invasive carcinoma (BRCA), pancreatic adenocarcinoma (PAAD), glioblastoma multiforme (GBM), and esophageal carcinoma (ESCA). The patients were divided into two groups based on the gene expression levels, where the top and bottom tertiles, in terms of gene expression, were considered as “high expressers” and “low expressers”, respectively.

**Fig. 7:**
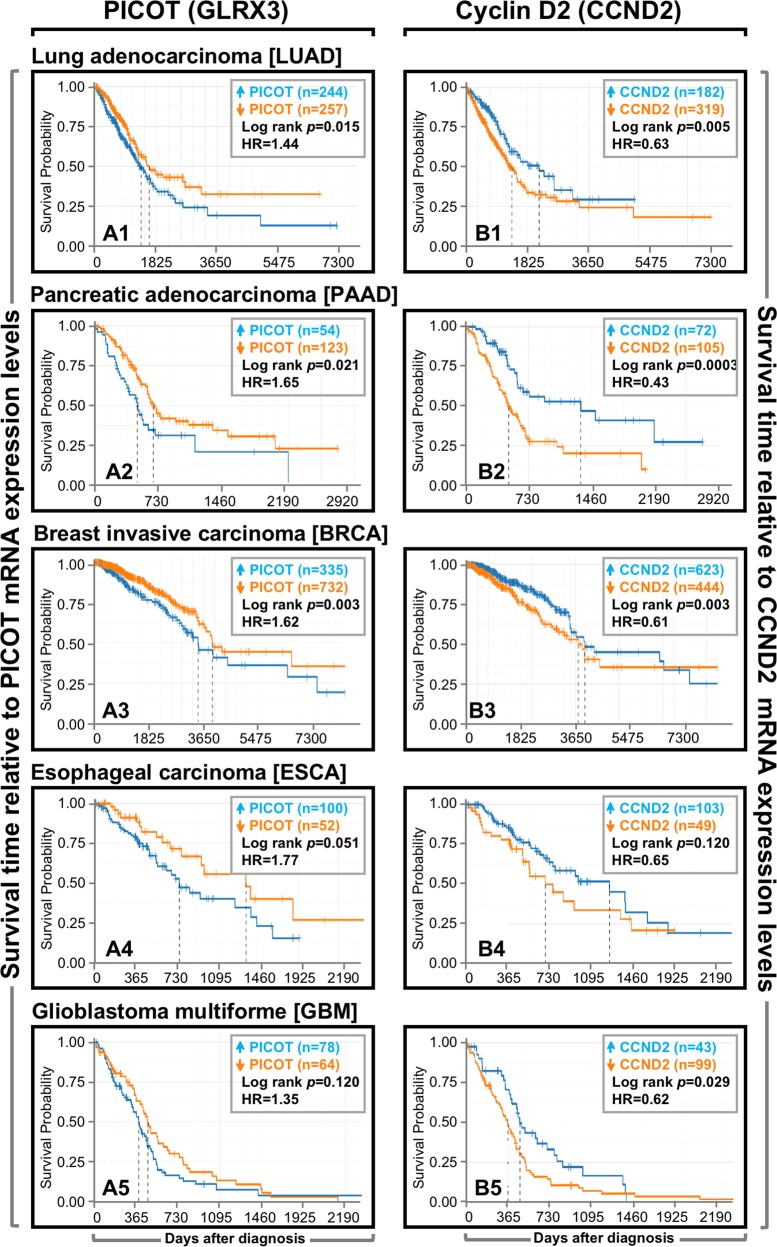
A relatively high expression of *PICOT* and low expression of *CCND2* correlate with poor patient survival in five different types of human cancers. *PICOT* (*Glrx3*) and *CCND2* mRNA expression and cancer patients’ clinical data were derived from the TCGA database. Patients were divided into high (blue color) or low (orange color) expression groups, using the maximally selected rank statistics that is implemented in the R package ‘survminer’. Survival of patients was visualized using the Kaplan–Meier estimator. The log-rank *p* value (used to estimate the significance of the differences) and the hazard ratio (HR) between the groups are shown in the inset of each panel

The Kaplan–Meier survival plots for gene expression vs. survival probability and the log-rank *p* value, as well as the hazard ratio in each type of tumors, were compared (Fig. 7). The results demonstrate a significant log-rank *p* value for *PICOT* in LUAD, PAAD, BRCA, and ESCA (Fig. 7A1–4). Similar tendency was observed for the log rank *p* value in GBM (Fig. 7A5), although the correlation with *PICOT* expression and survival probability (0.11) was statistically insignificant. Furthermore, a high hazard ratio (>1) in relation to *PICOT* expression was observed in all tumor patients. When the log rank *p* value was obtained for *cyclin D2* low vs. high expressers, it was statistically significant in LUAD, PAAD, BRCA, and GBM (Fig. 7B1–4), but not in ESCA (Fig. 7B5), and a low hazard ratio (<1) was observed in all tumor types. Absence of clinical data for the T-ALL patients did not allow to analyze the linkage between PICOT expression and patient survival in this cohort.

Thus, our results suggest that overexpression of *PICOT* and downregulation of *CCND2* correlate with poor overall patient survival in the majority of analyzed tumors.

## Discussion

The observation that PICOT deficiency results in embryonic lethality^4,5^ implicates PICOT in cellular functions that are critical for embryogenesis. In addition, PICOT knockdown led to a slower growth rate of Jurkat T cells (Fig. 6s) and impaired both cell cycle progression and multiplication of mouse fibroblasts and HeLa cells^4^. Furthermore, higher expression levels of PICOT were observed in T lymphocytes^11^ and cardiac muscle cells^8,26^ following their stimulation. Nevertheless, the molecular mechanisms by which PICOT affects cell growth and modulates cell cycle progression are not yet clear.

In a previous study, we demonstrated that PICOT can associate with EED and that both proteins can partially colocalize at the cell’s nuclei^13^. Furthermore, PICOT binding to EED had a positive effect on H3K27 methylation at the MYT1 PRC2 target gene^13^. Based on these findings, we suggested that PICOT binding to EED might have an impact on the assembly or coordination of the PRC2 protein subunits, modulate the EED-dependent EZH2-mediated histone H3 lysine 27 trimethylation, and contribute to the overall epigenetic regulation of chromatin silencing and remodeling.

In the present work, we found a first link between PICOT function and cell cycle regulation by demonstrating that PICOT binding to EED modulates the transcriptional regulation of the PRC2 target gene, *CCND2*, a cell cycle-regulating gene which encodes the cyclin D2 protein.

We demonstrated that PICOT associates with the chromatin-residing EED and that PICOT knock-down leads to a reduced H3K27me3 mark and a decrease in EED and EZH2 at the *CCND2* gene promoter. These events were accompanied by a marked rise in *CCND2* mRNA and protein expression. We hypothesize that increased levels of cyclin D2 might alter the activity of its target genes, such as *CDK4* and *CDK6*^27^, modulate the cell cycle progression^28^, and promote nuclear mechanisms leading to early embryonic cell cycle arrest^29^.

PICOT knockdown did not alter the global levels of H3K27me3 (not shown), as was demonstrated for other PRC2 auxiliary proteins, such as Jumonji and AT-rich interaction domain containing 2 (JARID2)^30^. A difference in the effect of PICOT knockdown on the two PRC2 target genes, *CCND2* and *HOXA2*, is not surprising, since knockdown of other PRC2 associated proteins, such as the Polycomb-like proteins (PCL), were also found to desilence only selected PRC2 target genes^20^.

EED is a core component of PRC2^17^, and its major biological activity is in connection with the PRC2 complex-mediated histone methyltransferase activity, which promotes epigenetic gene silencing. The critical role of EED in embryonic development was demonstrated in knock out mice that died on day ~12.5 post implantation^31–33^.

Along with EED, the PRC2 complex possesses three additional core components, namely zeste homolog 1 or 2 (EZH1/2), suppressor of zeste12 (SUZ12) and retinoblastoma protein-associated protein 46/48 (RbAp46/48). EZH1 and EZH2 are homologous proteins that serve as the PRC2 catalytic subunits that mediate the methyltransferase activity. While both proteins promote transcriptional silencing, they maintain repressive chromatin through different mechanisms^34,35^. Jurkat T cells express only a very small amount of EZH1 that resides in the cytoplasm, where it can interact with ZAP70^36^. In contrast, EZH2 which resides in the nucleus, is the predominant isoform in Jurkat T cells^37,38^ and its expression is associated with cell proliferation^34^.

EZH2 requires both EED and SUZ12 in order to maintain the PRC2 complex integrity and histone methyltransferase activity^23,39^. EED binding to EZH2 is mediated by the EED-WD40 domain^37^, and upon interaction with H3K27me3, EED recruits PRC2 complexes to established H3K27me3 and enforces the maintenance of the repressive epigenetic mark^17^.

Mammalian EED includes four distinct isoforms produced by alternative utilization of four distinct, in-frame translation start sites in the *Eed* mRNA^21,40^. Usage of the EED isoforms is regulated developmentally, suggesting that distinct EED isoform-containing PRC2 complexes may differ in their histone methyltransferase activity and/or target selectivity^21^. Differential association of PICOT with selected EED isoforms may thus have distinct effects on the activity and specificity of the PRC2-mediated trimethylation of H3K27.

The assembly, composition and biological activity of PRC2 complexes appear to be very complicated due to the potential interaction of the four core components of PRC2 with several additional auxiliary proteins^41,42^. A recent study indicated that PRC2 can associate with two alternative sets of proteins, namely PALI1/PCL1-3 or AEBP2/JARID2, which define two mutually exclusive antagonistic PRC2 subtypes, termed PRC2.1 and PRC2.2, respectively^43^. These two complexes exhibit divergent H3K27-methylation activities and regulate repression of different sets of polycomb target genes^43^. It is possible, therefore, that differential interaction of PICOT with PRC2.1-associated or PRC2.2-associated EED may have different impacts on cellular epigenetic mechanisms that alter distinct cell-specific gene expression signatures. While the nature of the EED isoforms that associate with each of the two PRC2 subtypes has not yet been determined, the existence of four distinct EED isoforms may add to the complexity and heterogeneity of this epigenetic mechanism.

Downregulation of *CCND2* mRNA was reported as a striking feature of several human tumors, including the lung^44^, breast^45,46^ and pancreas^47^ tumor tissues. This effect reflected hypermethylation at the *CCND2* gene promoter region which is rarely observed in NAT samples^44–47^. In agreement with these findings, we observed an increased hypermethylation at the *CCND2* promoter and reduced cyclin D2 expression in wild type Jurkat cells, whereas PICOT-deficient Jurkat cells exhibited reduced methylation and increased expression of cyclin D2.

Targeting of *CCND2* in non-small-cell lung carcinoma cells with the microRNA *miR-146a-5p*, inhibited cell cycle progression and cell proliferation^48^, and other *CCND2* targeting *miRs* inhibited the proliferation of additional types of cancer cells^49–51^. It appears, therefore, that downregulation of CCND2 is required for cell cycle progression and replication of certain cell types, and that overexpression of PICOT can lead to excessive downregulation of CCND2 expression, which might promote the cell growth in several types of human cancers.

The increased expression of PICOT protein in a variety of cancers, including Hodgkin’s lymphoma^11^ and breast^7^, colon^12^, and lung carcinoma^12^, encouraged us to test whether the negative correlation between PICOT and CCND2 expression, which was observed in Jurkat T cells, also exists in human cancers. Information obtained from the Cancer Genome Atlas (TCGA) database verified the negative correlation between *PICOT* and *CCND2* in eight different human cancer types.

More important, high expression of *PICOT* and low expression of *CCND2* correlated with poor patient survival in five different types of human cancers, namely lung and pancreatic adenocarcinoma, glioblastoma, and breast and esophageal cancer. Similar findings were observed in an independent validating dataset, using the Kaplan–Meir plotter, a web tool that reports difference in survival in an independent compendium of expression profiles from cancer patients (not shown). Interestingly, the existence of outliers in different cancer types (Fig. 6) suggests that additional mechanisms, besides PICOT-induced altered trimethylation of H3K27 at the *CCND2* gene promoter, are likely to affect the cell cycle and growth rate of these cells.

The above information may imply that high expression of PICOT and low expression of CCND2 are advantageous to the growth of transformed cells in selected tissues, while other or additional factors affect the growth of transformed cells in other tissues.

## Materials and methods

### Reagents and antibodies

Mouse monoclonal antibodies (mAbs) specific for PICOT were from Santa Cruz Biotechnology, Inc. (Santa Cruz, CA). Mouse anti-β-actin mAbs and normal mouse IgG were from Merck Millipore (Darmstadt, Germany). Mouse anti-Histone H3 mAbs and anti-Flag mAbs and rabbit anti-hemagglutinin (HA) and anti-RNA polymerase II (Pol II; Ab26721) polyclonal Abs were from Abcam Biotechnology Company (Cambridge, UK). Mouse anti-Flag mAbs (clone M2) were from Sigma-Aldrich, Israel. Rabbit anti-EED polyclonal Abs (Ab4469), which were used for Western blot analysis, and rabbit anti-EED Abs (09–774), which were used for ChIP-qPCR assay (09–774) were from Abcam and Merck Millipore, respectively. Rabbit mAbs anti-EZH2, anti-SUZ12, anti-trimethyl histone H3 (Lys 27, H3K27me3), anti-cyclin D2 and normal rabbit IgG were from Cell Signaling Technology Inc. (Danvers, MA). Horseradish peroxidase (HRP)-conjugated goat anti-mouse and anti-rabbit immunoglobulin Abs were from Abcam Biotechnology. Protein G-Sepharose®, Fast Flow (P3296) was from Sigma-Aldrich, Israel. Cyanine 3 (Cy3)-conjugated goat anti-mouse IgG and Cyanine 5 (Cy5)-conjugated goat anti-rabbit IgG Abs were from Jackson ImmunoResearch Laboratories, Inc*. (*West Grove, PA) and from Kirkegaard and Perry Laboratories (KPL), respectively. DAPI was from Biotium, Inc. (Hayward, CA). The full-length *PICOT* cDNA with a human influenza hemagglutinin (*HA*) tag in the pEF mammalian expression vector (*HA-PICOT*) has been previously described^1^. The *EED*_2-441_ cDNA introduced into the *pFLAG-CMV2* eukaryotic expression vector (*FLAG-EED*) and a mock plasmid (*FLAG-CMV*) were gifts of Dr. Dieter Adam (University of Kiel, Germany)^19^.

### Preparation of PICOT-deficient Jurkat T cell sublines

*PICOT* shRNA plasmids were designed and synthesized by SuperArray Bioscience (Frederick, MD) and introduced into an expression vector (pGeneClip) that directs the synthesis of small hairpin RNAs (shRNAs) in mammalian cells under the control of the polymerase III promoter, and contains the neomycin resistance gene for selection of stably transfected cells. We used a set of four sense strands of 21 nucleotide sequences of human *PICOT* (CCAACATACCCTCAGCTGTAT; CTACCCAGCGCTAATGAACAT; GTGGAAATTCTTCACAAACAT; ACTCCCTCAAGTTTC ATTTGT), plus a control scrambled sequence. The sense strand was followed by a 10-nucleotide spacer (CTTCCTGTCA) and a reverse complement of the same 21 nucleotide sequence. After cloning these short sequences downstream of the U1 promoter of the plasmid, the resulting pGeneClip-*PICOT*-shRNA plasmids were transfected into Jurkat E.6 cells by electroporation using a BioRad Gene Pulser. G418 resistant stable transfectants were isolated and cloned and their PICOT expression levels were determined by Western blot and qRT-PCR.

### Cell lines and culture conditions

The Jurkat human T cell line (clone E6.1; ATCC^®^ TIB-152^TM^), PICOT-deficient Jurkat-derived T cell lines (Jurkat.1A, Jurkat.2G), a control Jurkat cell line expressing a scramble *PICOT* plasmid (Jurkat.5A), THP1 human acute monocytic cell line (ATCC^®^ TIB-202^TM^), MOLT-4 human T lymphoblast cell line (ATCC^®^ CRL-1582^TM^), U-937 histiocytic lymphoma cell line (ATCC^®^ CRL-1593.2^TM^) and K-562 human myelogenous leukemia cell line (ATCC^®^ CCL-243^TM^) were maintained at a logarithmic growth phase in complete RPMI (RPMI-1640 supplemented with 10% heat-inactivated fetal calf serum (FCS), 2 mM L-glutamine, 50 units/ml penicillin, 50 μg/ml streptomycin (all from Biological Industries, Beit Haemek, Israel), and 0.5 μM β-mercaptoethanol (Sigma)). COS-7 epithelial kidney cells, which are derived from an African green monkey and express the Simian Vacuolating Virus 40 (SV40) TAg (ATCC^®^ CRL-1651^TM^), A-549 human lung carcinoma cell line (ATCC^®^ CCL-185^TM^), MDA-MB 453 human breast metastatic carcinoma cell line (ATCC^®^ HTB-131^TM^), MCF-7 human breast adenocarcinoma cell line (ATCC^®^ HTB-22^TM^) and HEK-293T human embryonic kidney cells, which express the SV40 Tag, were maintained in complete Dulbecco’s modified Eagle medium (DMEM; supplemented with 10% heat-inactivated FCS, 2 mM L-glutamine, 50 units/ml penicillin, 50 μg/ml streptomycin and 0.5 μM β-mercaptoethanol).

### Transient cell transfection

COS-7 cells that were maintained at a logarithmic growth phase were collected by trypsin treatment, washed in supplement-free DMEM, resuspended at 2 × 10^6^ cells/ml in 5 ml DMEM with 10% FCS and cultured in Cellstar^®^ tissue culture flasks (Greiner Bio-One 690170). Within 24 h of plating, adherent cells were transfected with the indicated DNA (5 µg/group) using polyethylenimine (PEI, Polysciences Inc.) at a ratio of 3:1 (PEI: total DNA) and analyzed after two additional days in culture.

### Preparation of cell lysates

Cells were resuspended in a lysis buffer (25 mM Tris-HCl, pH 7.5, 150 mM NaCl, 5 mM EDTA, 1 mM Na_3_VO_4_, 50 mM NaF, 10 µg/ml each of leupeptin and aprotinin, 2 mM AEBSF and 1% Triton X-100) followed by 30 min incubation on ice and centrifugation at 13,000 rpm for 30 min at 4 °C. Equal volumes of 2× sodium dodecyl sulfate (SDS) sample buffer were added to whole cell lysates (WCL) followed by vortexing, boiling for 5 min, and sample fractionation by SDS polyacrylamide gel electrophoresis (PAGE).

### Electrophoresis and immunoblotting

Samples containing whole cell lysates, chromatin lysates, or Ab immunoprecipitates were resolved by electrophoresis on 10 or 12.5% polyacrylamide gels using Bio-Rad Mini-PROTEAN II cells. Gel proteins were electroblotted onto nitrocellulose membranes (Schleicher and Schuell) at 100 V for 1 h, using BioRad Mini Trans-Blot transfer cells. After 1 h of membrane blocking with 3% BSA in TBS at 37 °C, the membranes were incubated with the indicated primary Abs followed by extensive washings in TBS and incubation with HRP-conjugated goat anti-mouse or goat anti-rabbit IgG Abs. Immunoreactive protein bands were visualized using the enhanced chemiluminescence (ECL) detection system followed by autoradiography.

### Chromatin immunoprecipitation and real-time qPCR

Chromatin immunoprecipitation (ChIP) was performed according to a published protocol^52^, with few modifications. Cells were crosslinked using 1% formaldehyde (Sigma-Aldrich, Israel) for 10 min at room temperature, washed twice in cold PBS, and then lysed by resuspension in 1 ml lysis buffer (20 mM Tris-HCl, pH 8.0, 85 mM KCl, 0.5% NP-40 and 1% protease inhibitor cocktail) for 10 min on ice. Nuclear pellets obtained by centrifugation (5 min, 5000 rpm, 4 °C) were resuspended in 200 μl nuclei lysis buffer (50 mM Tris-HCl, pH 8.0, 10 mM EDTA, 1% SDS and 1% protease inhibitor cocktail) for 10 min on ice, and samples were sheared by 5 rounds of 6 cycles of 30 s on/30 s off at a high-power setting of the Bioruptor sonicator (Diagenode), to yield input DNA enriched fragment sized 200–1000 bp.

The dimension of the chromatin fragments was determined empirically by agarose gel electrophoresis and ethidium bromide staining. Samples were spun down (20 min, 13,000 rpm, 4 °C) and the soluble chromatin fractions were collected, diluted (1:5) in a dilution buffer (0.01% SDS, 1.1% Triton X-100, 1.1 mM EDTA, 20 mM Tris-HCl, pH 8.0, and 167 mM NaCl) and incubated with protein G-sepharose beads for a preclearing step. Precleared lysates were incubated with bead-mobilized specific Ab or nonspecific isotype control Ab and kept overnight on a rotator at 4 °C. The following day, the beads were sequentially washed with buffer-1, buffer-2, and buffer-3, and twice with buffer-4.

Buffers used are listed as below:

Buffer-1: 0.1% SDS, 1% Triton X-100, 2 mM EDTA, 20 mM Tris-Cl, pH 8.0, 150 mM NaCl in ddH_2_O

Buffer-2: 0.1% SDS, 1% Triton X-100, 2 mM EDTA, 20 mM Tris-Cl, pH 8.0, 500 mM NaCl in ddH_2_O

Buffer-3: 0.25 M LiCl, 1% NP40, 1% deoxycholate, 1 mM EDTA, 20 mM Tris-Cl, pH 8.0 in ddH_2_O

Buffer-4: 10 mM Tris-HCl, 8.0 in 1 mM EDTA

Precipitated DNA fragments were eluted from the protein G sepharose beads using elution buffer (1% SDS, 50 mM NaHCO_3_, 140 mM NaCl, 10 mM Tris-HCl, pH 8.0, 1 mM EDTA). Subsequently, eluted DNA was treated with RNAase (0.2 µg/µl) at 37 °C for 1 h, followed by incubation with proteinase K at 65 °C for 5 h. The DNA was then purified using PCR clean-up kit (Macherey*-*Nagel kit) and amplified by real-time qPCR (40 cycles (Cycling stage: 15 s 95 °C/60 s 60 °C; melt curve stage: 15 s 95 °C/60 s 60 °C/30 s 95 °C/60 s 60 °C)) with primers specific for the indicated target genes. The forward and reverse primer sequences used in the RT-PCR experiments were as follows:

*CCND2* ChIP-qPCR forward primer: TAGGATCCGTTTTGAAGAAGCC, reverse CATTCTGTAGGTGTAGCACGCC^53^; *HOXA2* ChIP-qPCR forward primer: AGGAAAGATTTTGGTTGGGAAG, reverse AAAAAGAGGGAAAGGGACAGAC^54^, *ATF3* ChIP-qPCR forward primer: CTACAGTCACCTTGCGGTGC, reverse GAGGCTGGGAAGGGTAATGG^55^; *CDC6* ChIP-qPCR forward primer: GATTCCCTCCCCCGTTCA, reverse primer: CAATGAGAGAGCCCCAAGTCTT^53^; GAPDH ChIP-qPCR forward primer: TACTAGCGGTTTTACGGG CG, reverse TCGAACAGGAGGAGCAGAGAGCGA. Commercial primers for the *α-Satellite* were obtained from Cell signaling (#4486).

ChIP qPCR quantification was carried out as the percentage of the input chromatin. The relative enrichment was calculated by dividing the DNA amount obtained using the specific Ab, by the DNA amount obtained using the isotype control, for any of the targeted primers^54^.

$\% \;{\mathrm{of}}\;{\mathrm{Input}} = 100 \times 2^{\left( {{\mathrm{adjusted}}\;{\mathrm{input}} - {\mathrm{Ct}}\;\left( {{\mathrm{IP}}} \right)} \right)}$; where adjusted input = raw input Ct−log2 of dilution factor.

Average results were obtained from two independent ChIP experiments that were analyzed by three independent PCRs (*N* = 6 wells).

### Protein-protein chromatin immunoprecipitation

Protein-protein ChIP was performed according to a published protocol with few modifications^56^. Cell cross-linking and chromatin lysates were prepared as described in the ‘chromatin immunoprecipitation and real-time qPCR’ section. The soluble chromatin fraction was diluted (1:5) in dilution buffer (0.01% SDS, 1.1% Triton X 100, 1.1 mM EDTA, 20 mM Tris-HCl, pH 8, 167 mM NaCl) and incubated with protein G-sepharose beads for a preclearing step. Precleared lysates were incubated with bead-mobilized specific or nonspecific isotype control Ab and kept overnight on a rotator at 4 °C. The following day, beads were washed once using buffer-1, buffer-2, buffer-3 and twice with buffer-4, as indicated previously. After the last wash, 180 µl of sample buffer were added to the pellet and samples were kept at 95 °C for 30 min. The samples were then centrifuged and the supernatants (20 µl) were resolved by SDS-PAGE and analyzed by immunoblot.

### Cell staining and confocal microscopy

Cells (1.5 × 10^6^) were seeded on poly-L-lysine-coated 8 well µ-slide (ibidi Ltd.) and deposited on the slides by centrifugation at 1200 rpm for 5 min. Cells were fixed in 4% paraformaldehyde/PBS for 15 min at room temperature, permeabilized with PBS/0.2% Triton X-100 for 5 min and incubated with mouse anti-PICOT mAbs and rabbit anti-EED polyclonal Abs in a blocking buffer (PBS containing 1% BSA) for 1 h at room temperature. After three washes in PBS, the cells were incubated with Cy3-conjugated goat anti-mouse Ig Abs (1:500), Cy5-conjugated goat anti-rabbit Ig Abs (1:500), and DAPI (1 µg/ml) in PBS for 1 h at room temperature in the dark. Confocal microscope analysis was performed using the Olympus FluoView® FV1000 laser scanning confocal microscope.

### RNA extraction and quantitative PCR

For quantitative PCR analysis, RNA was extracted from Jurkat and Jurkat-derived sublines using an RNA isolation kit (YRB100, RBC Bioscience Corp.) according to the manufacturer’s instructions. For cDNA synthesis, 1 µg of RNA was reverse transcribed (RT) with the Bio-RT first strand cDNA synthesis kit (Bio-Lab) using random hexamer primers. The reaction was carried out for 60 min at 37 °C and terminated by incubation at 75 °C for 15 min. Real-time (RT)-PCR was performed on the ABI PRISM™ 7500 (Applied Biosystems™, Foster City, CA), using ABsolute qPCR SYBR Green kit (ABgene Inc., Portsmouth, NH) according to the manufacturer’s instructions. The RT-PCR reaction contained 50 ng of cDNA product and 500 nm of each of the primers. Primers for each gene studied were designed with overlapping exon boundaries, using the NCBI-BLAST software, and obtained from Sigma-Aldrich, Israel. All primers were designed to have <150 bp, to ensure high amplification efficiency. The forward and reverse primer sequences used for the RT-PCR amplification were as follows: *CCND2* forward primer: GGACATCCAACCCTACATGC, reverse CGCACTTCTGTTCCTCACAG; *SDHA* forward primer: ACAACTGGAGGTGGCATTTCTA, reverse TAATTTTCTAGCTCGACCACGG.

All experiments were performed in triplicates to ensure reproducibility. The cycling conditions included a polymerase activation step at 95 °C for 10 min, followed by 40 cycles of 95 °C for 15 s and 60 °C for 60 s. After PCR amplification, the specificity of the PCR was confirmed by melting temperature determination of the PCR product. Amplification efficiencies were verified by serial dilution standard curve fit. Relative quantification was determined by the ^ΔΔ^Ct method^57^, standardized against succinate dehydrogenase (*SDHA*), as a reference gene, and expressed as fold change.

### Tumor tissue and normal adjacent tissue data collections

Cancer patients’ clinical outcome and RNA expression data from tumor tissues and normal adjacent tissues were downloaded from the Cancer Genome Atlas (TCGA) data portal at the ISB cancer genomics cloud (https://isb-cgc.appspot.com/data)^58^ on July 15, 2018. For gene expression data, we selected the level-3 RNA-Seq-V0 data (Illumina GA or HiSeq sequencing platforms). RNA-Seq expression level read-counts were normalized, using FPKM values (Fragments per Kilobase of transcript per Million mapped reads), as provided in the downloaded files. Additional information regarding the data and analysis methods used in the processing of TCGA data can be found at https://docs.gdc.cancer.gov/Data/Bioinformatics_Pipelines/Expression_mRNA_Pipeline.

### Patients’ survival analysis

For every gene analyzed, patients were classified according to the high or low expression of the gene of interest. The classification was performed by calculating a mean cut of point using the MaxStat function (which is based on the maximally selected rank statistics)^59^, as available in the R package survminer (https://cran.rstudio.com/web/packages/survminer/index.html). For plotting the survival of the low and high expression patient groups the Kaplan–Meier estimator^60^ was used.

### PICOT-CCND2 correlation analysis

*PICOT* and *CCND2* mRNA expression levels in solid human cancers were compared using the TCGA dataset and the Pearson correlation. Outlier samples in total population were identified using quantiles, and samples from the upper and lower 0.1 quantiles were removed from the actual analysis. To analyze for potential correlation between PICOT and CCND2 expression in T-ALL, we searched the Gene Expression Omnibus (GEO) repository and searched for T-ALL data series. The dataset selected (GSE62156) had 64 sample, which were downloaded and processed using an R script inspired by the GEO2R option. Probe set 207506_at was used for PICOT and 200951_s_at was used for CCND2.

### Statistical evaluation

Statistical evaluation for sample comparison was carried out using the Prism5 software (GraphPad, La Jolla/CA, USA). Statistical tests used are indicated in the respective figure legends.

Statistical evaluation for TCGA datasets was carried out using R, version 3.4.1 (http://www.r-project.org). Patients’ clinical outcome data were analyzed using the *survival* package and plotted as Kaplan–Meier survival curves.

Unless specified otherwise, *p* values smaller than 0.05 were considered significant. An exact *p* value was calculated where applicable.

## Supplementary information

PICOT binding to chromatin-associated EED negatively regulates cyclin D2 expression by increasing H3K27me3 at the CCND2 gene promoter
