# Supplementary material for: PICOT binding to chromatin-associated EED negatively regulates cyclin D2 expression by increasing H3K27me3 at the CCND2 gene promoter
Source: Cell Death Dis. 2019 Sep 17;10(10):685. doi: 10.1038/s41419-019-1935-0 (PMC6746821; doi:10.1038/s41419-019-1935-0)
Supplement: Supplementary file 1 — PICOT binding to chromatin-associated EED negatively regulates cyclin D2 expression by increasing H3K27me3 at the CCND2 gene promoter [file 41419_2019_1935_MOESM1_ESM.docx]

**Supplementary Information**

**Table Is**

**Pearson’s correlation co-efficient values comparing TCGA dataset-derived *PICOT* and *CCND2* mRNA expression levels in different human cancers.**

| **Cancer** | **Total sample (N)** | **Sample remain after outlier removal (N1)** | **Pearson’ Correlation value (Cor)** | ***p* value** |
| --- | --- | --- | --- | --- |
| TCGA-PRAD | 481 | 477 | -0.28547 | 2.14E-10*** |
| TCGA-LUAD | 510 | 506 | -0.26826 | 8.67E-10*** |
| TCGA-PAAD | 177 | 173 | -0.32800 | 1.06E-05** |
| TCGA-GBM | 143 | 139 | -0.26973 | 0.001322* |
| TCGA-BRCA | 1071 | 1063 | -0.09714 | 0.001519* |
| TCGA-STAD | 373 | 369 | -0.15659 | 0.002557* |
| TCGA-ESCA | 152 | 148 | -0.21626 | 0.008292* |
| TCGA-OV | 354 | 350 | -0.13085 | 0.014293* |
| TCGA-UVM | 77 | 73 | -0.22238 | 0.058628 |
| TCGA-DLBC | 47 | 43 | -0.27237 | 0.077229 |
| TCGA-TGCT | 133 | 129 | -0.15164 | 0.086254 |
| TCGA-THCA | 497 | 493 | -0.06874 | 0.127433 |
| TCGA-COAD | 451 | 447 | -0.06666 | 0.159412 |
| TCGA-READ | 162 | 158 | -0.11057 | 0.166665 |
| TCGA-UCEC | 537 | 533 | -0.05887 | 0.174735 |
| TCGA-KIRC | 526 | 522 | -0.04918 | 0.262001 |
| TCGA-ACC | 79 | 75 | -0.10371 | 0.37591 |
| TCGA-THYM | 119 | 116 | -0.05298 | 0.572197 |
| TCGA-HNSC | 495 | 491 | -0.02314 | 0.608987 |
| TCGA-PCPG | 175 | 171 | -0.03797 | 0.621933 |
| TCGA-CHOL | 36 | 33 | -0.08818 | 0.625552 |
| TCGA-LUSC | 496 | 492 | -0.02061 | 0.648355 |
| TCGA-MESO | 81 | 77 | -0.02631 | 0.820333 |
| TCGA-KIRP | 287 | 283 | -0.00276 | 0.963139 |
| TCGA-SKCM | 103 | 99 | 0.026308 | 0.796034 |
| TCGA-KICH | 65 | 61 | 0.096230 | 0.460668 |
| TCGA-CESC | 296 | 292 | 0.055069 | 0.348403 |
| TCGA-BLCA | 405 | 401 | 0.058513 | 0.242377 |
| TCGA-SARC | 258 | 254 | 0.078143 | 0.214547 |
| TCGA-LGG | 499 | 495 | 0.081401 | 0.070376 |
| TCGA-UCS | 56 | 52 | 0.122220 | 0.388051 |
| TCGA-LIHC | 369 | 365 | 0.190740 | 0.000247** |

Abbreviations: TCGA, The cancer genome atlas; PRAD, Prostate cancer adenocarcinoma; ;LUAD, Lung adenocarcinoma; PAAD, Pancreatic cancer; GBM, Glioblastoma; BRCA, Breast cancer; STAD, gastric adenocarcinoma; ESCA, Esophageal cancer; OV, Ovarian cancer; UVM, Uveal melanoma; DLBC, Lymphoid neoplasm diffuse large B-cell lymphoma; TGCT, Testicular germ cell tumors; THCA, Thyroid cancer; COAD, Colon adenocarcinoma; READ, Rectum adenocarcinoma; UCEC, Uterine corpus endometrial carcinoma; KIRC, Kidney renal clear cell carcinoma; ACC, Adrenocortical carcinoma; THYM, Thymoma; HNSC, Head and neck squamous cell carcinoma; PCPG, Pheochromocytoma and paraganglioma; CHOL, Cholangiocarcinoma; LUSC, Lung squamous cell carcinoma; MESO, Mesothelioma; KIRP, Kidney renal papillary cell carcinoma; SKCM, Skin cutaneous melanoma; KICH, Kidney chromophobe; CESC, Cervical squamous cell carcinoma; BLCA, Bladder urothelial carcinoma; SARC, Sarcoma; LGG, Low grade glioma; UCS, Uterine carcinosarcoma; LIHC, Liver hepatocellular carcinoma; Signficance was estimated using the t-test for the signficance of the correlation coefficient. For simplicity, the following indicators are used: *** = p<1E-6; ** = p<1E-3; * = p<0.05.


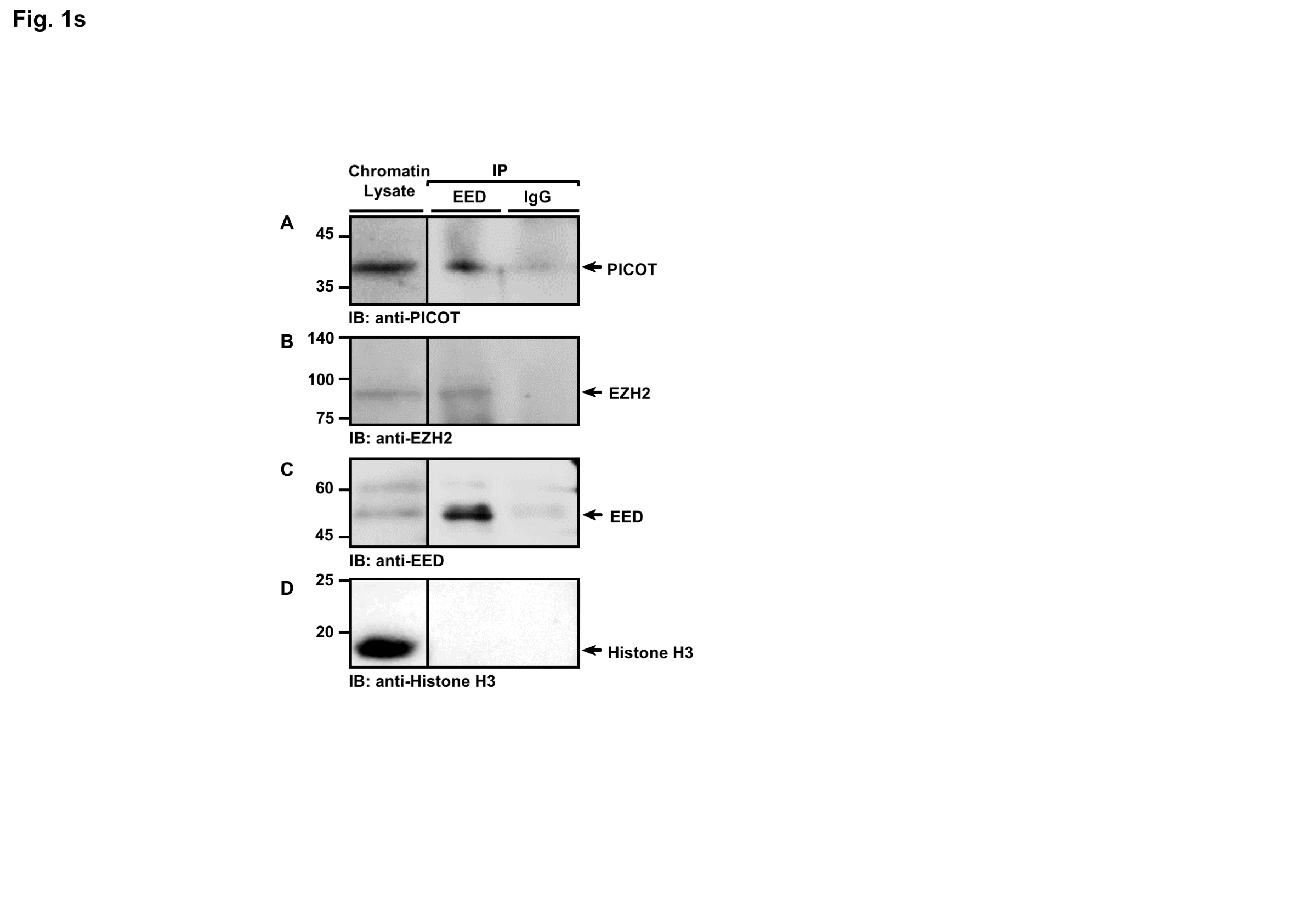


**Figure 1s. PICOT and EZH2 co-immunoprecipitate with EED from Jurkat T chromatin lysates.**

Jurkat cells (20×10^6^) were fixed with 1% formaldehyde for 10 min at room temperature and chromatin was isolated using the protein-protein ChIP protocol. Chromatin lysates were precleared with protein G-sepharose beads, and lysate samples (equivalent to 10^7^ cells per group) were incubated overnight at 4°C with protein G sepharose bead-imobilized rabbit anti-EED (Ab4469) Abs or rabbit normal IgG. Proteins were eluted from the beads by boiling (30 min) in 180 µl β-mercaptoethanol-containing sample buffer and centrifuged. Supernatants (20 µl) were subjected to SDS-PAGE on 10% gel under reducing conditions. Chromatin lysates (~1.25 µg/lane) were boiled and electrophoresed in parallel. Proteins were then electroblotted onto a nitrocellulose membrane, immunoblotted with mouse anti-PICOT mAbs followed by immunoperoxidase ECL detection system and autoradiography (A). Membranes were sequentially immunoblotted with rabbit anti-EZH2 mAbs (B), rabbit anti-EED polyclonal Abs (C), and mouse anti-Histone H3 mAbs (D). Anti-EED immunoblot served as a loading control for immunoprecipitation and anti-Histone H3 immunoblot served as a marker for chromatin lysates. Rabbit IgG light chain-specific mouse mAbs were used to detect a specific EED isoform. The position and identity of specific protein bands is indicated by arrows. IP, immunoprecipitation; IB, immunoblot.


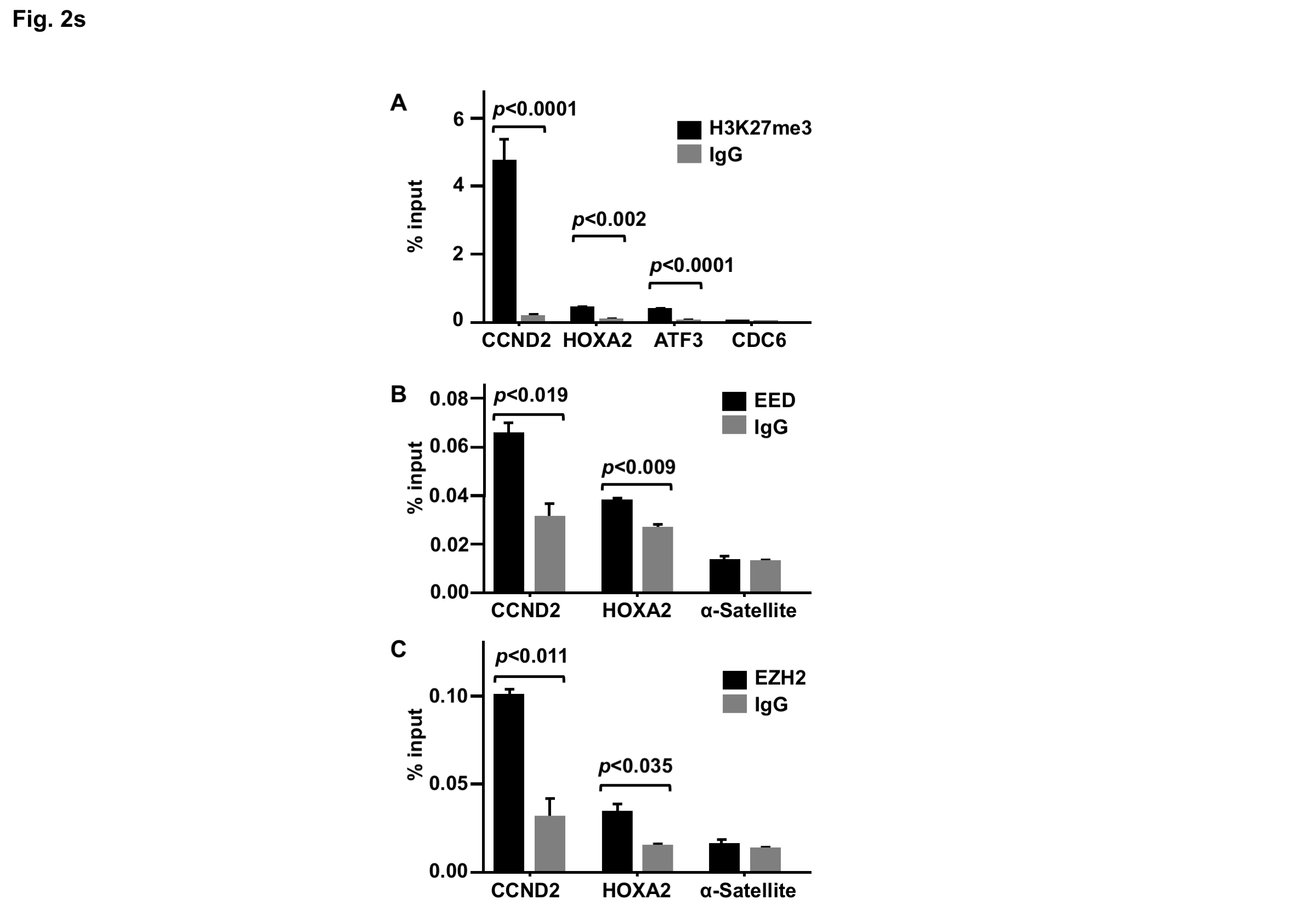


**Figure 2s. Optimization of ChIP-qPCR analysis for anti-H3K27me3, anti-EZH2 and anti-EED Abs using Jurkat chromatin lysate.**

Jurkat cells (20x10^6^) were fixed in 1% formaldehyde for 10 min at room temperature, lysed and sonicated. Chromatin lysates (10^7^ cell equivalent/group) were precleared on protein G-sepharose beads and then incubated overnight at 4°C with protein G-sepharose bead-immobilized rabbit anti-H3K27me3 mAbs (A), rabbit anti-EZH2 mAbs (B), rabbit anti-EED Abs (09-774) (C) or normal IgG. After overnight incubation on a rotator, the beads were washed sequentially with low-salt buffer, high-salt buffer, LiCl buffer and Tris-EDTA buffer. The DNA was eluted from the immune complexes (and from crude sonicated chromatin lysates) using 10% w/v Chelex. Isolated DNA was used as a template for RT-qPCR for the indicated PRC2 target genes. ChIPs using normal rabbit IgG served as a negative control. *CDC6* and *α-satellite* were used as control, PRC2 non-target gene. ChIP enrichments relative to total input signal were calculated and values were presented as mean percentage ± s.e.m for two independent analyses. Each analysis was carried out in triplicate wells. The level of significance was determined using the t-test with the help of GraphPad Prism 7 software (GraphPad, La Jolla/CA, USA).

**Figure 3s. ChIP-qPCR analysis demonstrating the PICOT protein does not directly associated with *CCND2* gene promoter.**

Jurkat cells (**A)** or COS-7 cells that were transiently transfected with HA-PICOT vector **(B)** were fixed using 1% formaldehyde for 10 min at room temperature. Next chromatin lysates were proceeded for ChIP-qPCR analysis using mouse anti-PICOT mAbs **(A)** or rabbit anti-HA polyclonal Abs **(B)**, as described in material and methods. Precipitated DNA was eluted, purified and analysed using real-time PCR. Specificity of rabbit anti-HA mAbs was determined by Western blot analysis **(C).** ChIPs with protein G-sepharose beads served as a negative control. ChIP enrichments were calculated relative to total input signal and expressed as a mean percentage ± standard error mean of the calculated values. T-test was calculated using GraphPad Prism 7 software to determine the level of significance between two groups (specific Abs *vs*. bead).


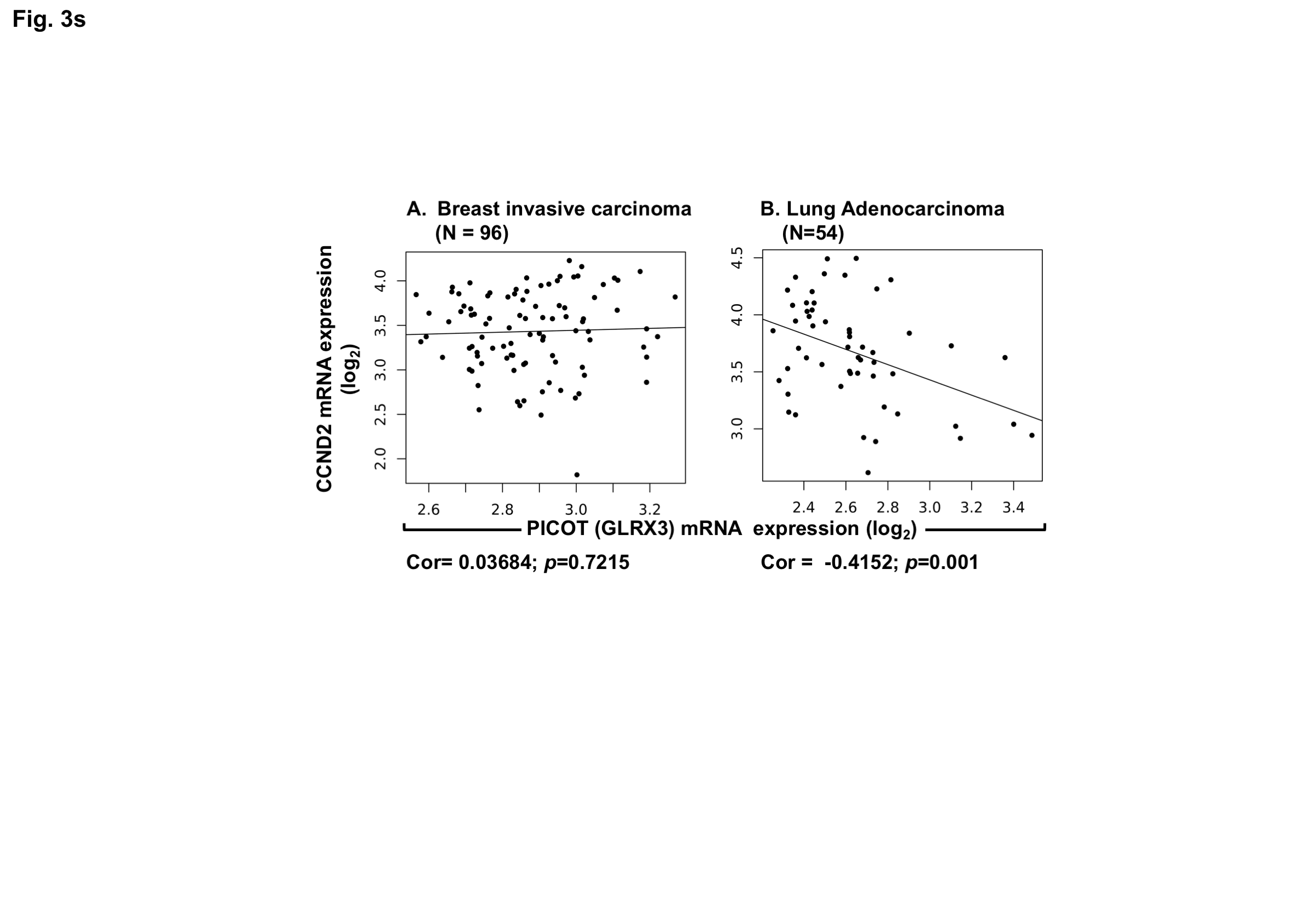


**Figure 4s. Pearson’s correlation analysis between PICOT and CCND2 mRNA expression levels in tumor-adjacent normal tissues.**

*PICOT* (*Glrx3*) and *CCND2* mRNA expression levels in two types of tumor-adjacent normal human tissues were obtained from the TCGA using ISB Cancer Genomics Cloud. To avoid effects from outlier samples, the top and bottom deciles of gene expression were removed from the analysis. The correlation between *PICOT* and *CCND2* mRNA expression at log2 scale is illustrated as scatter plot. Each dot represents a single tissue sample. For each type of tissue, the total number of patients (N) obtained, the Pearson’s correlation value (Cor), and the *p* value are indicated.


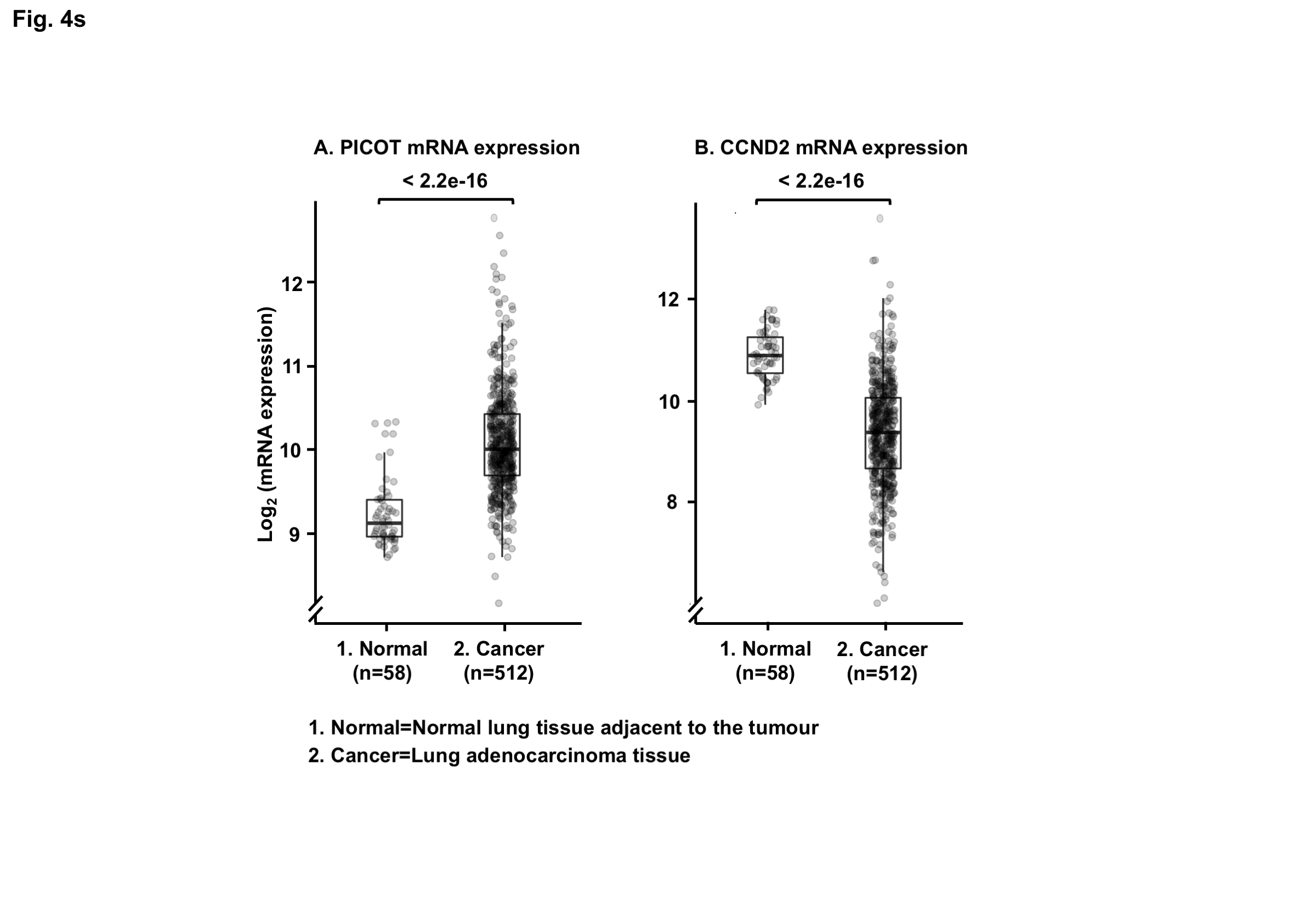


**Figure 5s. Human lung adenocarcinoma has higher expression levels of PICOT and lower expression levels of CCND2 compared to the mRNA expression levels in adjacent normal lung tissue.**

TCGA-derived mRNA expression levels of *PICOT* (*Glrx3*, **A**) and *CCND2* (**B**) genes in lung adenocarcinoma versus the normal adjacent tissue were obtained from the ISB Cancer Genomics Cloud and presented as box plots. X axis represents the tissue type (lung adenocarcinoma vs. the adenocarcinoma-adjacent, normal lung tissue) and Y axis represents log2 mRNA expression of the corresponding gene. For each tissue type, the individual values for each sample are indicated as grey circles. Statistical analyses of mRNA expression levels between the tumor tissue and the normal, tumor-adjacent tissue were conducted using a Student’s *t*-test between groups, and their significance (*p* values) are presented.

**Figure 6s. Stable expression of PICOT-shRNA down-regulates the in vitro growth rate of Jurkat T cells.** Jurkat T cell clones that stably express PICOT-shRNA or a control scrambled RNA (PICOT-scRNA) were seeded at a density of 5x10^3^/200μl/well in wells of 96-well microtiter plates. Cell proliferation was measured using the Cell Proliferation Kit II (XTT; Roche), according to the manufacturer’s instructions. XTT was added for the final three hours of culture and the colorimetric reading of the formazan dye at 450 nm was carried in an ELISA plate reader at the indicated time intervals. Background absorbance of each sample at 630 nm was subtracted from the readings at 450 nm. The results represent the average of triplicate samples with error bars showing the standard error of the mean (SEM).
